# Intersectional masculinism: how patriarchal power adapts across regime changes in Indonesia

**DOI:** 10.3389/fsoc.2026.1897725

**Published:** 2026-10-29

**Authors:** Sopian Tamrin, A. Octamaya Tenri Awaru, Syamsidah Syamsidah, Bastiana Bastiana

**Affiliations:** State University of Makassar, Makassar, Indonesia

**Keywords:** Crenshaw, discontinuity without transformation, Foucault, Indonesia, intersectional masculinism, siri’, women’s political exclusion

## Abstract

Globally, women’s political empowerment remains a resistant frontier of gender equality, particularly in sub-national executive positions. This study introduces intersectional masculinism as a theoretical framework to explain the durability of women’s political exclusion in executive positions, moving beyond static patriarchy accounts. Drawing on 6-month ethnographic fieldwork in Bone Regency, Indonesia, a site maintaining an absolute male executive monopoly for seven decades despite meeting legislative quotas, we identify three adaptive configurations of masculine dominance: feudal-aristocratic (kingdom era), military-bureaucratic (New Order), and capitalist-electoral (Reform era). Synthesizing Foucault’s analytics of discontinuity and technologies of the self with Crenshaw’s intersectionality, we demonstrate that Bugis siri’ (honor/shame) operates as an intersectional technology of the self that disciplines women’s political subjectivity through spatial confinement, internalized self-regulation, and differential operation across class and lineage. Our findings reveal a pattern of “discontinuity without transformation”: while political regimes shift radically from kingdom to republic, from authoritarianism to democracy, male executive monopoly persists through reconfiguration of privilege-conferring intersections (lineage, military affiliation, economic capital). This study contributes a transferable framework for understanding durable political exclusion across the Global South, from Pakistan’s izzat to the Philippines’ maratabat and Turkey’s namus. We argue that democratic transitions alone cannot dismantle gendered monopolies if underlying intersectional barriers remain unchallenged.

## Introduction

Bone Regency, the largest Bugis territory in South Sulawesi, presents a compelling political landscape. Long before the contemporary discourses of political modernization and gender equality gained prominence, the Bone Kingdom (14th–20th centuries) witnessed significant female political leadership. Of the 32 recorded reigns, six were held by women, accounting for approximately 18.75% of the total leadership periods (Bahri et al., 2023). From We Banrigau (1470–1,510) to We Fatimah Banri (1871–1895), women occupied the highest echelons of political authority. However, since the dissolution of the Bone Kingdom in 1950, the regency has been governed by 13 regents, all of whom have been men. In the five direct regional head elections conducted since 2003, not a single woman has ever appeared as a candidate for regent or deputy regent (KPUD Bone, 2025).

This is not merely a quantitative observation; it constitutes an absolute executive monopoly that has persisted for over seven decades. This condition persists despite the regency meeting the 30% legislative quota for women’s representation (13.33% in, 2024) and possessing a Gender Development Index (93.63) that exceeds the provincial average (BPS, 2024). Bone, therefore, cannot be reduced to a case of low representation; rather, it represents a paradox in which increasing legislative representation of women has not correlated with access to executive power, which remains a domain of masculine exclusion.

Globally, political inequality constitutes the most resistant dimension of gender disparity. The Global Gender Gap Report 2025 indicates that while Educational Attainment (94.9%) and Health and Survival (86.2%) have nearly achieved parity, Political Empowerment languishes at only 22.5% (World Economic Forum, 2025). Merely 27.3% of parliamentary seats worldwide are occupied by women, and the proportion of female heads of state or government remains below 10% (ANFREL, 2024). In Asia (22.1%) and the Middle East-North Africa region (16.7%), representation is even lower. In Southeast Asia, normative regression has occurred: between 2014 and 2022, social norms concerning gender equality deteriorated, with a 15% increase in the belief that higher education is more important for men and a 14% increase in the acceptance of intimate partner violence (OECD, 2024). Indonesia reflects this pattern: despite enacting the UU TPKS = Undang-Undang Tindak Pidana Kekerasan Seksual (Sexual Violence Crime Law) in 2022 and raising the minimum age of marriage, the perception that employment should be prioritized for men increased by 12%, while acceptance of domestic violence surged from 36 to 55% (OECD, 2024). In this context, Bone emerges as a unique case within both regional and national patterns, in which democratic transition has failed to dismantle masculine dominance in the executive sphere.

Why does Bone, which historically produced female rulers, now fail to generate a single female regency candidate? The extant literature has not adequately addressed this question because of three principal limitations. First, most studies on women’s political participation in Indonesia focus on legislative representation and the implementation of the 30% quota policy (Ardiansa, 2016; Aulya et al., 2022; Bintari, 2022), thereby neglecting the executive domain where policymaking authority is concentrated. Bone shows that a regency can meet legislative quotas while maintaining an absolute male monopoly over the executive, a disparity that remains undertheorized. Second, existing research tends to treat patriarchy monolithically, as a uniform and unchanging structure (Nimrah and Sakaria, 2015). This approach obscures how patriarchal power transforms across historical periods, adapting to novel political and economic conditions. Third, although scholars have begun to acknowledge intersectional factors such as class and ethnicity (Nimrah and Sakaria, 2015; Collins and Bilge, 2020), no systematic analysis has examined how these identity layers interact with cultural discourse, specifically siri’ (honor/shame) and associated Bugis values in shaping women’s political subjectivity and their access to executive power. Anthropological studies have documented the dynamism of siri’ (Prasetio et al., 2025; Baharuddin et al., 2025), yet its operation as a political technology of the self remains insufficiently theorized.

To address these gaps, this study poses three research questions: (1) How have the configurations of political actors in Bone shifted across the three eras—the kingdom, the New Order, and the Reform era—and how have these shifts affected women’s access to executive power? (2) What cultural mechanisms, specifically the discourse of siri’ and its associated values (malebbi, warani, pesse), operate to discipline women’s bodies, limit their mobility, and shape their political subjectivity? (3) How do gender, class (noble/non-noble), and economic capital intersect to produce patterns of inclusion and exclusion that cannot be adequately explained by any single factor?

This study offers three principal contributions. First, theoretically, we introduce the concept of intersectional masculinism to analyze how male dominance adapts across regime changes. Unlike static conceptions of patriarchy, this concept captures how masculine power reconfigures itself as feudal-aristocratic (kingdom era), military-bureaucratic (New Order), and capitalist-electoral (Reform era) while preserving the male executive monopoly. This extends hegemonic masculinity theory by specifying the intersectional mechanisms (gender+lineage, gender+military, gender+capital) through which dominance is reproduced. Second, methodologically, we synthesize Foucault’s analytics of discontinuity and technologies of the self with intersectionality into a replicable protocol comprising genealogical mapping, discourse analysis of cultural texts, ethnographic tracking of internalized self-regulation, and an intersectional matrix that traces how discourse operates differently across class and lineage. Third, in contextual terms, we provide the first systematic analysis of executive exclusion in Bone Regency. This regency meets legislative quotas yet maintains an absolute male executive monopoly for seven decades, including no female regent candidate in five direct elections. By examining how Bugis siri’ operates as a political technology, this study offers comparative insights for Southeast Asian contexts where cultural honor codes intersect with democratic institutions to reproduce exclusion.

### Literature review

#### Gender, power, and political exclusion: mapping contemporary debates

This review maps the key theoretical and empirical debates informing this study’s examination of women’s political marginalization in Bone Regency, Indonesia. Organized around three interconnected trajectories, (1) theoretical synthesis of Foucault and intersectionality, (2) recent empirical studies of Indonesian women’s political participation, and (3) anthropological scholarship on Bugis gender dynamics, the review identifies critical gaps this study addresses. We argue that existing research underutilizes how cultural discourses and structural barriers intersect to produce durable exclusion and lacks historical discontinuity analysis in studies of Indonesian gender politics. We address these gaps through the Foucault–Crenshaw synthesis operationalized below.

### Literature review

#### Gender, power, and political exclusion

This literature review maps the key theoretical and empirical debates that inform this study’s examination of women’s political marginalization in Bone Regency, Indonesia. Departing from previous presentations that tended to compartmentalize studies into discrete sub-sections, this review organizes the discussion around three interconnected thematic intersections, explicitly identifying the critical gaps this research addresses.

##### Women’s political participation in Indonesia: from quotas to implementation

Research on women’s political participation in Indonesia over the past decade has developed along several trajectories. Most studies focus on legislative representation and the implementation of the 30% quota policy (Ardiansa, 2016; Aulya et al., 2022; Bintari, 2022). These studies have documented both the progress and the limitations of the quota approach: while the number of women in parliament has increased, the quality of representation, the extent to which women genuinely advocate for women’s interests, remains contested (Bintari, 2022). Recent research on the, 2024 legislative election (analyzing nearly 10,000 candidates) found that while voters tend to penalize female candidates, political parties can effectively counteract these biases through strategic list placement. However, parties simultaneously nominate far fewer women than men and apply different selection criteria, while high levels of party system fragmentation decrease the odds of women being elected (Muharam et al., 2024).

Research on election supervision bodies documents persistently low representation: among 1,907 commissioner positions across 514 districts, women hold only 14%, far below the 30% quota (Muharam et al., 2024). The Westminster Foundation for Democracy (2025) introduces the concept of “the domestication of women in politics,” noting that 45% of women parliamentarians are concentrated in social and domestic portfolios, while women hold only 14.5% of standing committee leadership roles. This pattern of “managed inclusion” reveals how “new forms of exclusion emerge to preserve existing hierarchies” as women’s representation grows.

However, these studies share a common limitation: they focus on national legislative representation, neglecting the local executive sphere where policymaking power is concentrated. A regency may satisfy legislative quotas while simultaneously maintaining an absolute male monopoly over the executive, a disparity that remains undertheorized. This study addresses this gap by focusing on district-level executive exclusion in Bone.

##### Masculinity and politics in Indonesia

Research on masculinity in Indonesian politics has begun to move beyond static patriarchy frameworks toward more dynamic analyses. Wulan (2024), in her study of the, 2024 presidential campaign, reveals how “familial values” continue to function as markers of ideal male leadership, suggesting that Indonesian masculinities are characterized by “restrained masculinities” rather than aggressive dominance. This finding challenges assumptions that political masculinity in Southeast Asia is always articulated through aggression or authoritarianism; rather, restrained masculinities emphasizing wisdom, composure, and family responsibility can constitute a more subtle yet equally effective form of dominance.

Research on post-conflict masculinities in Aceh, Maluku, and Mindanao demonstrates how “masculine elites” maintain dominance in decision-making through adaptive strategies that reconfigure rather than dismantle patriarchal power (Southeast Asia Peace Forum, 2024). These studies indicate that transitions from conflict to democracy do not automatically produce gender redistribution; rather, masculine elites exploit transitional moments to consolidate their positions through new formations such as religious-based leadership or economic authority.

Although these insights are valuable, research on political masculinity in Indonesia has not systematically analyzed how masculine dominance reconfigures itself across radical regime changes—from kingdom to republic, from authoritarianism to democracy. In other words, these studies tend to focus on a single historical period, so they fail to capture the pattern of “discontinuity without transformation” that constitutes the core of this study’s findings. Moreover, analysis of how masculinity operates intersectionally across class, lineage, and economic capital remains limited.

##### Bugis gender dynamics

Anthropological scholarship on Bugis gender has moved beyond static descriptions of siri’ (honor/shame) toward analyses of contemporary transformation. Baharuddin et al. (2025) document “a slow transition toward gender elasticity” in Bugis families, propelled by education, economic engagement, and contact with external ideas of equality. Women increasingly participate in financial decisions and leadership roles, while men engage more in domestic responsibilities. However, “cultural values of malebbi (modesty) and warani (courage) still influence gender ideals,” creating intergenerational conflict as tradition is renegotiated rather than abandoned.

Prasetio et al. (2025) examine shifts in siri’ interpretations in response to modernity, finding a shift from strict pre-marital modesty toward a symbolic understanding compatible with modern lifestyles. Studies of Bugis women’s representation in cultural production reveal persistent subordination, with women caught between conventional and Muslim ideologies (Rauf et al., 2024).

However, these anthropological studies focus on family, ritual, and cultural production rather than formal politics. The critical gap is the absence of systematic analysis of how siri’ operates as a political technology that regulates women’s access to public space and shapes political subjectivity. This study bridges this divide by analyzing siri’ through a Foucauldian lens as a technology of the self that disciplines female bodies and produces patriarchal subjectivity, while attending to its intersectional operations across class and lineage. Awaru et al. (2026) further explore the experiences of unmarried women in Bugis culture, showing how cultural norms around marriage and honor continue to shape women’s subjectivities and social positions.

##### Research gaps

When the three research traditions—political participation, studies of masculinity, and Bugis anthropology—are placed alongside one another, three interconnected gaps emerge:

First, the executive gap. Studies of women’s political participation in Indonesia largely focus on legislative representation and quota implementation at the national level, while overlooking the local executive sphere where policymaking power is concentrated. Bone presents a puzzle that existing frameworks cannot explain: a regency that meets legislative quotas yet maintains an absolute male executive monopoly for over seven decades. This is not merely “underrepresentation”; this is total exclusion that demands a different theoretical explanation.

Second, the historical gap. Research on political masculinity in Indonesia, while increasingly dynamic, has not systematically analyzed how masculine dominance reconfigures itself across radical regime changes from kingdom to republic, from the Old Order to the New Order, from authoritarianism to democracy. Studies on post-conflict masculinities (Southeast Asia Peace Forum, 2024) demonstrate that transitions do not automatically produce gender redistribution, but they do not offer a framework for analyzing long-term adaptation across multiple regime changes. Bone, with its long history from kingdom to democracy, provides a natural laboratory for such analysis. Our finding of “discontinuity without transformation” that political form changes while masculine monopoly persists offers a transferable pattern for understanding durable exclusion in the Global South.

Third, the discursive-intersectional gap. Anthropological studies of Bugis have documented siri’ as a dynamic cultural value, and studies of political participation have begun to acknowledge intersectional factors such as class and ethnicity. However, these two traditions have not been synthesized to analyze how siri’ operates differently across intersections—for noble *versus* commoner women, for women with *versus* without political family backgrounds—in shaping political subjectivity. Consequently, the mechanisms through which cultural discourse translates into political exclusion remain a “black box” in the literature.

##### Why Foucault–Crenshaw integration is necessary

These gaps—executive, historical, and discursive-intersectional—demand a theoretical framework that can simultaneously (1) trace power shifts across historical discontinuities, (2) analyze how discourse produces docile subjects, and (3) explain how these processes operate differently across social positions. This is precisely where the integration of Foucault and Crenshaw becomes analytically necessary, not merely optional.

Foucault’s analytics of discontinuity (Foucault, 1972) enable the tracing of how patriarchal power reconfigures itself across regime changes, a need that Crenshaw’s structural intersectionality alone cannot address. Foucault’s concept of technologies of the self (Foucault, 1988) provides the tools to analyze how siri’ operates as internalized self-regulation, not merely external constraint. Foucault’s concept of biopower (Foucault, 1977) explains how women’s bodies become repositories of collective honor.

To fully appreciate the analytical purchase of Foucault’s framework, it is necessary to outline several core Foucauldian concepts that underpin our analysis. First, Foucault’s concept of power/knowledge posits that power and knowledge are mutually constitutive: knowledge is not neutral but is produced within relations of power, and power operates through the production of truth. Second, discourse, in Foucault’s sense, refers to historically situated systems of statements that construct objects, subjects, and practices; discourse is not merely linguistic but material and productive. Third, disciplinary power operates through techniques of surveillance, normalization, and spatial regulation that produce docile bodies. Fourth, panopticism describes a modality of power in which the possibility of constant surveillance induces self-regulation. Fifth, episteme denotes the historical *a priori* that defines the conditions of possibility for knowledge in a given era. These concepts collectively inform our analytical framework: they allow us to trace how siri’ operates as a discursive formation that produces gendered subjects, how spatial confinement and self-regulation discipline women’s bodies, and how shifts in episteme across eras reconfigure the legitimating principles of masculine dominance while maintaining patriarchal outcomes.

However, Foucault’s analytics do not attend to how discursive production operates differently across social positions, a critique advanced by feminist theorists (McNay, 2013). This is where Crenshaw’s framework of intersectionality (Crenshaw, 1989; Collins and Bilge, 2020) becomes crucial. Intersectionality ensures attention to how the same discourse siri’ produces different political subjectivities for noble *versus* commoner women, and for women with *versus* without political families (to mapparenta). Intersectionality also enables analysis of how which intersections matter shifts across eras: lineage in the kingdom era, military affiliation in the New Order, and economic capital in the Reform era.

Why Foucault rather than alternative theoretical perspectives? While Bourdieu’s concept of symbolic violence illuminates how domination is misrecognized, it does not provide the genealogical tools to trace discursive ruptures across historical eras. Gramsci’s theory of hegemony explains cultural consent but lacks Foucault’s microphysics of power and attention to technologies of the self. Feminist institutionalism focuses on formal rules and norms but often neglects the productive role of discourse in shaping subjectivity. Foucault’s unique contribution lies in his synthesis of historical discontinuity, discursive production, and technologies of the self, which together enable us to analyze how masculine power adapts across regime changes while producing self-regulating gendered subjects. This is precisely the analytical insight needed to explain the “Bone Paradox” of persistent male executive monopoly despite radical political transformations.

Thus, the integration of Foucault and Crenshaw enables us to pose a question that neither framework alone can address: How does the same siri’ discourse produce different political subjectivities for women in different social positions, and how do these configurations of power adapt across regime changes while maintaining executive exclusion?

##### Contributions of this study

This study bridges the three gaps identified above through:

Systematic executive analysis: Unlike previous studies that focus on the legislature, this study examines Bone’s absolute executive monopoly over seven decades, shifting attention from “quantities of representation” to “quality of access to power.”

Historical discontinuity analysis: Unlike masculinity studies that tend to focus on a single period, this study traces changes across multiple historical eras. By tracing the configuration of political actors across three eras (kingdom, New Order, and Reform), this study reveals a pattern of “discontinuity without transformation” that has implications beyond Bone.

Discursive-intersectional analysis: Unlike anthropological studies that focus on family and ritual and political studies that focus on formal institutions, this study analyzes siri’ as an internalized political technology that operates differently across class and lineage, thereby bridging the division between “culture” and “politics” that has constrained existing literature.

## Method

### Paradigm and research approach

This study employs a qualitative approach grounded in a Foucauldian interpretive paradigm, utilizing the concepts of discontinuity and episteme as both analytical tools and methodological guides (Foucault, 1972). History is not treated as linear progression but as a series of discursive ruptures in which regimes of truth shift, thereby enabling analysis of how patriarchal power transforms across political eras while maintaining its core. An instrumental case study design (Stake, 2005) is adopted, with Bone Regency serving as the research site, selected for its historical contrast: six female rulers in the kingdom era (1470–1895) stand in stark contrast to the absolute male executive monopoly since 1950, including the absence of any female candidate in five direct elections (KPUD Bone, 2025). This contrast renders Bone a revelatory case (Yin, 2018) for examining how cultural-political discourse, particularly siri’ (honor/shame), adapts across regimes to reproduce masculine hegemony. The design, informed by a critical feminist perspective (Collins and Bilge, 2020), enables genealogical tracing of discursive formations that produce and legitimize women’s exclusion.

### Participants and recruitment procedures

Forty-two participants were recruited through purposive and snowball sampling over a 6-month period (September 2025–January 2026), encompassing nine categories: female politicians (7), male politicians/public officials (2), women’s organization activists (7), academics/researchers (6), cultural/traditional Figures (7), religious leaders/faith-based organizations (2), community/village leaders (5), journalists (2), and general activists/students (4).

The inclusion criteria were formulated explicitly for each category to ensure participants had direct and relevant knowledge of Bone’s political processes. Female politicians comprising six members of the Bone DPRD = Dewan Perwakilan Rakyat Daerah (Regional People’s Representative Council); for the, 2024–2029 period (Andi Tenri Walinonong from Gerindra, Sulfiana from Gerindra, Andi Purnama Sari Amier from Gerindra, Andi Nurjaya from PKS, Yuyun Andriyani from PKS = Partai Keadilan Sejahtera (Prosperous Justice Party); and Hj. Adriani A. Page from PPP = Partai Persatuan Pembangunan (United Development Party) and one former DPRD member/activist (Andi Asia Pananrangi) were included for their direct experience as political actors and their insights into structural and cultural barriers confronting women. Male politicians/public officials included a KPU = Komisi Pemilihan Umum (General Elections Commission) Bone commissioner and a political observer involved in candidate recruitment and selection processes. Women’s organization activists were recruited based on their engagement in gender advocacy, women’s protection, and empowerment, with a minimum of 5 years of experience in Bone and active participation in policy advocacy networks. Academics/researchers were those with publications or research on South Sulawesi who had actively conducted research in Bone for at least 3 years. Cultural/traditional figures were recognized by their communities as holders of traditional knowledge, capable of understanding siri’ values, and active in preserving Bone’s cultural heritage. Religious leaders/faith-based organization figures were leaders or administrators of Islamic organizations at the regency level and were active in religious and community forums. Community/village leaders were village heads or respected informal figures in their communities involved in village deliberations. Journalists were reporters covering politics and events in Bone for at least 5 years with access to political and bureaucratic sources. General activists/students were those engaged in youth organizations or non-governmental organizations (NGOs) concerned with sociocultural or political issues.

Of the 42 participants, 22 were women and 20 men, ranging in age from 17 to 60 years (mean age, 42.3 years). Fourteen participants were from noble backgrounds (anak arung) and 28 from non-noble backgrounds (ata), based on informant self-identification and genealogical verification through interviews with traditional figures. This diversity of backgrounds enriches the depth and complexity of the issues obtained.

Sampling reached thematic saturation at the 36th interview, when additional interviews no longer generated new themes or codes; an additional six interviews (totaling 42) were then conducted for negative case analysis to test and refine emerging thematic boundaries (Guest et al., 2020).

### Data collection

Data were collected through four triangulated methods to enable reconstruction of discursive formations across eras (Flick, 2018; Denzin, 1978):

1. In-depth interviews. Semi-structured interviews were conducted for 60–150 min in Indonesian or Bugis, depending on participant preference. The interview guide covered three domains: (a) participants’ political trajectories, including how they entered politics and the obstacles encountered; (b) understanding of cultural values (siri’, malebbi, warani, pesse); and (c) perceptions of gender representation and power in Bone. All interviews were recorded with participant consent and transcribed verbatim.
2. Focus group discussions (FGDs). Four FGDs were conducted with 6–8 participants per group, grouped by category (FGD 1: female politicians; FGD 2: activists and academics; FGD 3: cultural and religious figures; FGD 4: community leaders and journalists). FGDs aimed to capture collective sense-making and meaning negotiation among informants regarding themes such as the legitimacy of female leadership and changing gender norms.
3. Participant observation. Observation was conducted in various arenas: political meetings and campaigns, community gatherings and traditional ceremonies, and public spaces such as cafes and markets where political discussions occur. Observational data were recorded in ethnographic field notes documenting non-verbal behavior, spatial interactions, and performative practices that reproduce or challenge gender norms.
4. Document analysis. The following documents were analyzed to trace discursive shifts genealogically: (a) historical texts such as the La Galigo epic; (b) cultural texts such as pappaseng proverbs; (c) contemporary documents including party decrees, election records, and election reports from KPUD Bone; and (d) policy reports from DP3A Bone and BPS.

### Triangulation and data analysis

To ensure the validity and credibility of findings, this study employed three-dimensional triangulation (Denzin, 1978; Flick, 2018). Source triangulation compared statements across informant categories; for example, claims about high political costs (>Rp15 billion) were validated not only by politicians but also by journalists, activists, and community figures. Divergent perspectives across sources were recorded as negative data to refine interpretations. Method triangulation compared interview findings with observational data and document analysis; for example, discourse on siri’ as a constraint on women’s spatial mobility expressed in interviews was compared with observed spatial practices in public spaces and with similar representations in pappaseng texts. Researcher triangulation involved two researchers independently coding 20% of the transcript data using the same coding scheme, achieving an inter-coder reliability rate of 87%, considered excellent for qualitative research (Miles et al., 2020). Coding discrepancies were discussed to reach consensus before applying codes to the remaining data.

Data analysis followed Braun and Clarke’s (2006, 2021) thematic analysis framework with an intersectional lens (Khanam et al., 2024), implementing a four-step protocol that is transparent and replicable:

Step 1 (Coding). All interview transcripts, FGD recordings, field notes, and documents were imported into NVivo 14. Line-by-line coding employed a hybrid approach: deductive codes derived from Foucault (e.g., “disciplinary spatialization,” “technology of the self”) and Crenshaw (e.g., “intersectional invisibility”), and inductive codes emerging from the data (e.g., “kosong-kosong,” “to mapparenta”).

Step 2 (Theme Development). Codes were aggregated into candidate themes following Braun and Clarke’s (2021) six-phase guide. Two overarching themes were identified: social barriers (historical configurations of political actors across eras) and cultural barriers (discursive construction of gender through siri’ and associated values), each containing six sub-themes derived from Foucauldian concepts (discontinuity, episteme, biopower, technologies of the self).

Step 3 (Discourse Mapping). For each theme, key discursive formations were mapped across the three political eras (kingdom, New Order, and Reform), including (a) identification of recurring statements and metaphors in cultural texts, (b) tracing their transformation across historical discontinuities, and (c) analysis of their performative effects (Butler, 1990) in producing gendered subjects.

Step 4 (Intersectional Matrix). Following Carastathis (2014), an intersectional matrix was constructed with gender as the primary axis, crossed with class (noble/non-noble), lineage (political family/non-political family), and economic capital (high/low). Each cell in the matrix was populated with data from informants occupying those intersectional positions, revealing how the same discourse (e.g., siri’) operates differently across social positions. Negative case analysis was conducted to test and refine thematic boundaries.

### Researcher reflexivity and positionality

This research is explicitly acknowledged as a non-neutral practice of knowledge production. The research team comprises Indonesian academics with complementary positionalities: a “familiar outsider” fluent in Bugis, and an “insider” with aristocratic connections, both identifying as young activists with extensive socio-political networks in Bone.

Identity as credible young activists provided access to political and cultural elites, enabling acquisition of candid testimonies regarding informal power mechanisms. However, this proximity carries the potential for affinity bias, the tendency to unconsciously favor elite narratives or overlook grassroots perspectives. To minimize bias, the research team used the following strategies: (a) daily reflexive journals, wherein each researcher recorded assumptions, emotional reactions, and their position vis-à-vis informants and findings; (b) cross-validation with informants from non-elite backgrounds (ordinary citizens, grassroots activists, and women from non-noble families); and (c) regular critical discussions among researchers to challenge emerging assumptions and ensure interpretations remained grounded in data rather than personal preferences.

We recognize that our access to spaces of power is shaped by our social capital as academic-activists, enabling us to capture the usually concealed “kitchen” of politics, which also means that lower-status informants may be more reluctant to speak openly in our presence. Accordingly, interviews with non-elite informants were conducted in neutral settings and facilitated by recruiters from their own communities to foster a sense of safety and trust.

### Ethical considerations and limitations

This study adhered to social research ethical protocols: all participants provided written informed consent after a comprehensive explanation of the research purpose, risks, and their right to withdraw at any time; participant identities were protected through coding and anonymization, except for public figures who explicitly consented to name disclosure; raw data (recordings and transcripts) were securely stored and accessible only to the research team; and the study obtained ethical approval from the researchers’ affiliated institution.

The study has four principal limitations. First, as a single case study, findings cannot be statistically generalized to other regencies; however, analytical generalization to similar Global South contexts remains possible (Yin, 2018). Second, the 6-month fieldwork duration may not capture long-term processes such as full electoral cycles or slow cultural transformation; future longitudinal research is needed. Third, access to political elites was sometimes constrained by availability and willingness to speak, potentially yielding “diplomatic” or “polished” narratives, which we addressed through source triangulation and follow-up interviews. Fourth, the researchers’ positionality as young activists with elite access, despite mitigation efforts, inevitably shapes data accessibility and interpretation, an inherent characteristic of qualitative research involving human interaction and power relations.

## Findings

### Sociocultural barriers to women’s political participation: an intersectional analysis

This section presents the findings from 6 months of ethnographic fieldwork in Bone Regency, including 42 in-depth interviews, four focus group discussions, and extensive document analysis. Drawing on Foucault’s concepts of discontinuity and discourse, and Crenshaw’s intersectionality, we organize our findings around two overarching themes: social barriers (examined across three historical eras) and cultural barriers (examined through discursive constructions of gender). Each finding is supported by direct quotations from informants, revealing how intersecting systems of power produce durable exclusion.

### Social barriers I: historical discontinuity and the persistence of elite masculine configurations

The history of political power in Bone Regency reveals a pattern of discontinuity without transformation. Although political regimes have shifted dramatically from kingdom to republic, from authoritarianism to democracy, male-dominated power configurations have adapted and persisted. Our analysis across three eras (kingdom, Old Order/New Order, and Reform) demonstrates that each transition produced new forms of masculine hegemony while maintaining women’s exclusion from executive power.

### Kingdom era (14th–20th century): feudal-aristocratic masculinity and the exception that proves the rule

Contrary to narratives of unbroken patriarchal tradition, the Bone Kingdom (1330–1951) featured significant female political leadership. Of 32 recorded reigns, 6 were held by women, accounting for approximately 18.75% of leadership periods. The first ruling queen, We Banrigau Mallajange ri Cina, led from 1,470–1,510, followed by We Tenri Patuppu (1602–1,611), We Bataritoja (1714–1715 and 1724–1749), We Imaniratu (1823–1835), We Tenriawaru (1857–1860), and We Fatimah Banri (1871–1895) (see Table 1).

**Table 1:** List of Bone Kingdom Rulers (1330–1951).

No.	Name/title	Reign period	Gender
1	La Ubbi Mata Silompoe (Manurunge ri Matajang)	1,330–1,365	Male
2	La Ummasa (Petta Panre Bessie)	1,365–1,368	Male
3	La Saliyu (Kerampelua)	1,368–1,470	Male
4	We Banrigau (Mallajange ri Cina)	1,470–1,510	Female
5	La Tenrisukki (Mappajunge)	1,510–1,535	Male
6	La Uliyo Bote-e (Matinroe ri Itterung)	1,535–1,560	Male
7	La Tenrirawe Bongngkange (Matinroe ri Gucinna)	1,560–1,564	Male
8	La Inca (Matinroe ri Addenenna)	1,564–1,565	Male
9	La Pattawé (Matinroe ri Bettung)	1,565–1,602	Male
10	We Tenri Patuppu (Matinroe ri Sidenreng)	1,602–1,611	Female
11	La Tenriruwa (Sultan Adam, Matinroe ri Bantaeng)	1,611–1,616	Male
12	La Tenripale (Sultan Abdullah, Matinroe ri Tallo)	1,616–1,631	Male
13	La Maddaremmeng (Matinroe ri Bukaka)	1,631–1,644	Male
14	La Tenriaji (Arungpone, Matinroe ri Pangkep)	1,644–1,672	Male
15	La Tenritatta (Daeng Serang, Petta Malampe-e Gemme’na, Arung Palakka)	1,672–1,696	Male
16	La Patau Matanna Tikka (Sultan Matinroe ri Nagauleng)	1,696–1714	Male
17	We Bataritoja (Datu Talaga Arung Timurung, Sultanah Zainab Zulkiyahtuddin)	1714–1715 and 1724–1749	Female
18	La Padassajati (Toappeware, Petta Rijalloe, Sultan Sulaeman)	1715–1718	Male
19	La Pareppa (Tosappewali, Sultan Ismail, Matinroe ri Sombaopu)	1718–1721	Male
20	La Panaongi (Topawawói, Arung Mampu, Karaeng Bisei)	1721–1724	Male
21	La Temmassongé (Toappawali, Sultan Abdul Razak, Matinroe ri Mallimongeng)	1749–1775	Male
22	La Tenritappu (Sultan Ahmad Saleh)	1775–1812	Male
23	La Mappasessu (Toappatunru, Sultan Ismail Muhtajuddin, Matinroe ri Lebbata)	1812–1823	Male
24	We Imaniratu (Arung Data, Sultanah Rajituddin, Matinroe ri Kessi)	1823–1835	Female
25	La Mappaseling (Sultan Adam Najamuddin, Matinroe ri Salassana)	1835–1845	Male
26	La Parenrengi (Arungpugi, Sultan Ahmad Muhiddin, Matinroe ri Ajang Bantaeng)	1845–1857	Male
27	We Tenriawaru (Petta Pancaitana Besse Kajuara, Sultanah Ummulhuda, Matinroe ri Majennang)	1857–1860	Female
28	La Singkeru Rukka (Sultan Ahmad Idris, Matinroe ri Topaccing)	1860–1871	Male
29	We Fatimah Banri (Datu Citta, Matinroe ri Bolampare’na)	1871–1895	Female
30	La Pawawói (Karaeng Sigeri, Matinroe ri Bandung)	1895–1905	Male
31	La Mappanyukki (Sultan Ibrahim, Matinroe ri Gowa)	1931–1946	Male
32	La Pabbenteng (Arung Macege, Matinroe ri Matuju)	1946–1951	Male

Six of the 32 rulers (18.75%) were women. All female rulers were of aristocratic lineage.

However, as cultural figure Andi Singkeru Rukka (47) explained, this female leadership was strictly circumscribed by class:

“In Bone’s tradition, leadership attaches only to blue blood. When a woman becomes Arung, it is first because she is ‘Anak Arung’ [child of a ruler], and only second because of personal capacity. Ordinary people viewed them as heavenly beings, not as representatives of women in general”.

This testimony illustrates Foucault’s concept of discontinuity: the kingdom era’s configuration of power, feudal-aristocratic masculinity, did not produce gender equality but rather a class-privileged exception that reinforced, rather than dismantled, patriarchal hierarchy (Foucault, 1972). This interpretation was reinforced by religious and cultural leader Rahmatunnair (52):

“It is true that several queens ruled Bone, such as We Tenriawaru and We Batari Toja. But it must be understood: they ascended the throne not because the kingdom opened broad selection spaces for all women. This was purely because they were daughters of the nobility at that time. The system remained very closed; power circulated only within the royal and noble families. Women from the common populace, no matter how capable, would never touch the palace”.

From an intersectional perspective (Crenshaw, 1989), this reveals how gender and class intersected to produce a “discrimination within discrimination”: noble women accessed power, but commoner women were excluded entirely. The presence of female rulers thus does not indicate gender equality but rather the operation of feudal-aristocratic masculinity, where class intersected with gender for a tiny elite. Gender studies scholar Prof. Safira Suhra (52) offered a critical feminist reading of this historical pattern:

“Historical data on women’s leadership in Bone is often raised as evidence of past equality. However, I think it’s important to examine their background and class. All six female leaders were aristocrats. Their leadership actually reinforced a hierarchical feudal system; it did not dismantle gender inequality or represent all strata”.

This confirms our theoretical claim: what we term feudal-aristocratic masculinity operates not through universal male dominance but through the intersection of gender and aristocratic lineage, allowing even noble women to access power in exceptional circumstances. In this configuration, power was not about gender *per se* but about blood, and blood was coded masculine except when no male heir existed.

These testimonies reveal a crucial finding: the presence of female rulers in the kingdom era does not indicate gender equality; rather, it demonstrates what we term feudal-aristocratic masculinity, a configuration in which power is legitimized through noble lineage, and women can access it only at the intersection of gender and aristocratic class. This pattern, as Crenshaw’s intersectionality would predict, created a “discrimination within discrimination” in which commoner women were excluded entirely.

### The Republic of Indonesia era (1950–1998): military-bureaucratic masculinity and the consolidation of male monopoly

The transition to the Indonesian republic marked a significant discontinuity in political form but not in gendered outcomes. From 1950 to 2003, all 15 regional heads (bupati) in Bone were men (see Table 2). Political scientist and observer Supriadi (49) explained the logic of this period:

**Table 2:** Women and men in Bugis culture: cultural lexicon and discursive functions.

Cultural term	Translation	Meaning	Practical relevance	Discursive function (power mechanism)
Women				
Lise Kowari	Room Contents	Women as inhabitants of rooms	Domestic	*Spatial confinement*—Produces women as interior, private, and needing protection
Malebbiq	Unpretentious, polite	The less they leave the house, the more malebbiq	Domestic	*Self-disciplining virtue*—Internalized norm linking morality to immobility
Malampe Nawa-nawa	Has good memory	Organizing contents of house	Domestic–Public (masculine)	*Cognitive domestication*—Female intelligence channeled into household labor
Ri olona Sumpangnge	At the front door	Women’s mobility limited to threshold	Domestic	*Threshold regulation*—Constructs home as women’s spatial boundary
Indo Botting	Mother of the bride	Arranging weddings, domestic matters	Domestic	*Reproductive role fixation*—Women’s social value tied to marriage and care
Mancujung	Uphold/carry burdens	Ideal symbol of women’s work in carrying	Public–Marginal	*Visible but peripheral labor*—Acknowledges work while excluding from authority
Men				
Mareso	Hard worker	Strong work ethic, discipline	Public	*Productive masculinity*—Legitimizes men through economic contribution
Passompe	Migrant worker	Men leave home to earn a living	Public	*Spatial expansion*—Men’s mobility as natural, necessary, and virtuous
Ri Cappa’na Linoe	At the edge of the world	Unlimited mobility and public access	Public	*Unbounded spatial legitimacy*—Men belong everywhere, women nowhere outside home
Ri Congari	Can be asked for opinions	Source of advice and guidance	Domestic–Dominant–Public	*Epistemic authority*—Men as knowledge-holders and decision-makers
Ri Sanresi	Responsible	Trustworthy, reliable, leadership	Domestic–Dominant–Public	*Guardianship mandate*—Men as protectors of women and family honor
La Mata Esso	The sun	Enlightening, intelligent, problem-solver	Domestic–Dominant–Public	*Illumination metaphor*—Men as natural leaders who “light the way”
La Pateddungi	Shading	Able to protect, nurture, guard	Domestic–Dominant–Public	*Protective patriarchy*—Men’s dominance framed as care and shelter
Mallempa	Carrying	Men carry more weight	Dominant–Public	*Burden justification*—Male authority legitimated by assumed greater responsibility

“Discursive function” column identifies how each cultural term operates as a Foucauldian technology of power producing gendered subjects through spatial regulation, epistemic authority, and internalized self-discipline. These are not mere descriptions but performative statements that construct the realities they name (Butler, 1990).

“Historically, in the New Order era, the rulers/ruling party prioritized figures from the military (ABRI) to occupy regional head positions. And we know they were, by definition, men. This historical trajectory certainly strengthened the patrilineal patron model”.

This exemplifies Foucault’s discontinuity without transformation: the political regime changed from kingdom to republic, and from Old Order to New Order, yet the gendered outcome of male monopoly persisted. What shifted was the legitimating principle: from noble lineage to military affiliation (Foucault, 1972). Muhammad Takbir (40), a sociocultural observer, elaborated on how feudalism transformed rather than disappeared:

“Feudalism in Bone Regency remains very strong; power is still contested by certain circles possessing genealogies of power from the past. After the pangadereng (customary system), no women have emerged as leaders. Practically, since the advent of democracy, women have at most appeared as speculative candidates, and even then not in mainstream conversations. They are discussed but never reach actual nomination”.

This testimony reveals the layering of configurations: military-bureaucratic masculinity did not replace feudal-aristocratic masculinity but merged with it. Military officers sent to Bone often had or strategically acquired genealogical ties to the aristocracy, creating a hybrid elite. Intersectionally, this meant that legitimate power required the simultaneous possession of male gender, military affiliation, and noble lineage (Carastathis, 2014).

The militarization of politics during the New Order (1966–1998) was particularly significant. As Supriadi noted, military officers, overwhelmingly men, were systematically placed in regional leadership positions. This created what we term military-bureaucratic masculinity: a configuration where legitimate power required the intersection of male gender, military affiliation, and state loyalty. This effectively excluded all women, since the military was an almost exclusively male institution.

This configuration effectively excluded all women, since the military was an almost exclusively male institution. As Enloe (2000) argues, militarization strengthens patriarchal values and limits women’s public sphere access. In Bone, this operated as an intersectional barrier: even a woman with noble lineage could not access power because she lacked the military affiliation that had become a prerequisite for executive office.

### Reform era (1999–present): capitalist-electoral masculinity and the new barriers of money politics

The transition to direct elections after 1999 raised hopes for more inclusive politics. However, five direct regional head elections (2003–2024) have produced zero female regents or deputy regents (see Tables 3, 4). Journalist Abd. Muhaimin (38) identified the central obstacle:

**Table 3:** Comparison of theoretical frameworks for analyzing masculine dominance.

Dimension	Hegemonic masculinity (Connell)	Hybrid masculinities (Bridges and Pascoe)	Intersectional masculinism (this study)
Core focus	Cultural ideal of male dominance	Men’s appropriation of marginalized masculinities to soften dominance	Adaptive configuration of privilege-conferring intersections (lineage, military, capital)
Treatment of power	Relational but hierarchical	Incorporation and softening	Layered and cumulative across historical discontinuities
Intersectional sensitivity	Weak (class/gender only)	Moderate (gender + race/class)	Core analytic (gender + lineage + military + capital as configurations)
Treatment of change	Slow transformation	Elastic accommodation	Discontinuity without transformation (form changes, male monopoly persists)

This table compares three theoretical frameworks for analyzing masculine dominance: hegemonic masculinity (Connell), hybrid masculinities (Bridges & Pascoe), and intersectional masculinism (proposed in this study). It highlights the distinctive focus of intersectional masculinism on layered privilege accumulation and discontinuity without transformation.

**Table 4:** Discontinuity without transformation across political eras.

Era	Political form	Legitimating principle	Privilege-conferring intersection	Gendered outcome
Kingdom	Monarchy	Noble lineage + spiritual charisma	Gender + Lineage	Female rulers only as a class exception
New Order	Authoritarian state	Military loyalty + state service	Gender + Military	Total female exclusion
Reform	Electoral democracy	Economic capital + patronage	Gender + Capital	Female legislators but no executive

The table demonstrates that while the “how” of power shifted, the “who” of power remained constant. This is discontinuity without transformation, the signature pattern of intersectional masculinism.

“Just winning a district legislative candidacy requires funds up to billions [of rupiah], let alone running for regent. Current contests are very thick with money politics. Political costs are increasingly expensive, compounded by communities being open to accepting all sorts of politically motivated gifts”.

This testimony illustrates Foucault’s concept of the episteme: the conditions of possibility for legitimate political power have shifted. In the Reform era, economic capital has replaced or supplemented noble lineage and military affiliation as the primary barrier to entry. This is not merely a practical constraint but a discursive formation that defines the “serious candidate” as one who can raise billions of rupiah (Foucault, 1972). Supriadi (49) provided more specific figures:

“Campaign costs for district legislative candidates range from 300-500 million rupiah, but can reach 1-1.5 billion if buying votes. Provincial candidates need 3-4 billion. For regional head elections, costs reach approximately 15 billion or higher”.

From an intersectional perspective, this creates a systematic barrier for women, who historically have less access to economic capital than men. The 15 billion rupiah threshold operates as an intersectional filter: only those at the intersection of male gender (or female gender with family wealth) and economic capital can enter the contest. This explains why the few women who succeed are almost exclusively dynastic successors (Collins and Bilge, 2020). Journalist Agung Purnomo (32) added regional variation:

“West Bone still relies on figures/reputation. So political costs aren’t too high, around 300-500 million. In the city, it’s purely a battle of political costs. Range 500 million to 1 billion, maybe even more. South Bone also relies on figures, North Bone is slightly contaminated by the city”.

Interestingly, Agung noted a gendered dimension to campaign financing:

“Women, actually, can be said to spend less than men. This is inseparable from the fact that most women who run are those who indeed have a big family name. Certainly they have their own base, so the costs incurred are more for operational needs”.

This seemingly positive observation that women spend less reveals the operation of what we term capitalist-electoral masculinity. Women enter not as independent political actors but as family successors whose “big family name” substitutes for personal capital. This is not a feminization of politics but a dynastic feminization of presence without feminization of power. As our intersectional framework predicts, gender alone does not determine access; gender intersects with lineage and economic capital to produce differentiated possibilities (Crenshaw, 1989).

Several female politicians confirmed this pattern. Sulfiana (30), a Gerindra DPRD member, explained that she ran at her family’s request after her uncle, a former Deputy DPRD Chair, passed away. ‘After discussions among the family, they decided I should run representing the family,’ she stated. This pattern of women entering politics as family successors was nearly universal among female politicians in Bone. This testimony operationalizes our concept of technologies of the self (Foucault, 1988): women do not autonomously choose to enter politics; they are positioned by family discourses that define their political role as familial duty rather than individual ambition. This internalized self-understanding that one runs “representing the family” rather than representing constituents or oneself is precisely how patriarchal power operates through subjectivity, not coercion.

Andi Tenri Walinonong (32), the first female Chair of the Bone DPRD, described her trajectory:

“Initially I applied to become a policewoman three times, but never passed. Finally, my family encouraged me to run for DPRD member in Bone, from electoral district IV. I first ran in 2019 through the Nasdem party, but wasn’t seated then even though I got quite a few votes. In the, 2024 legislative election, I ran again through the Gerindra party, and Alhamdulillah, I was elected. And I got the most votes for a Gerindra legislative candidate in Bone Regency, which was 7,828 votes.”

When asked about her support base, Andi Tenri acknowledged the importance of family networks:

“I have several relatives who are village heads in Ulaweng district, which is my electoral area. My mother was also a village head, and currently my brother is also still an active village head”.

This case is analytically revealing: Andi Tenri’s success is a textbook example of intersectional privilege. She is female, yet she succeeded because she sits at the intersection of gender (female), lineage (political family with multiple village heads), and what Bourdieu (1977) would call social capital (networks of relatives in formal positions). Her trajectory confirms our core finding: women can access legislative power when, and only when, they possess other privilege-conferring attributes that compensate for their gender. The executive sphere, however, remains closed because the intersectional threshold there is even higher, requiring not just lineage and social capital but massive economic capital (15 billion rupiah) that even elite women rarely control.

These findings reveal what we term capitalist-electoral masculinity: a configuration in which legitimate power requires the intersection of male gender (or female gender with family succession), economic capital, and patronage networks. The high cost of politics (15 billion rupiah for regent candidates) systematically excludes women without access to elite family wealth and networks. Even when women are elected, as in the six female DPRD members (13.33% of the legislature), they are almost exclusively successors to male political dynasties. The theoretical integration of intersectional masculinity provides a framework for examining how actors’ attributes intersect within different configurations of power. During the kingdom era, the primary intersections were gender, noble descent, and spiritual charisma. During the New Order, the intersection shifted to gender, military affiliation, and state loyalty. During the Reform era, the intersection became gender, economic capital, and patronage networks. This shift shows that masculine hegemony in Bone is dynamic and adaptive, always seeking new intersections that can maintain the dominance of certain male groups within changing political contexts (Christensen and Jensen, 2014; Figure 1).

**Figure 1:**
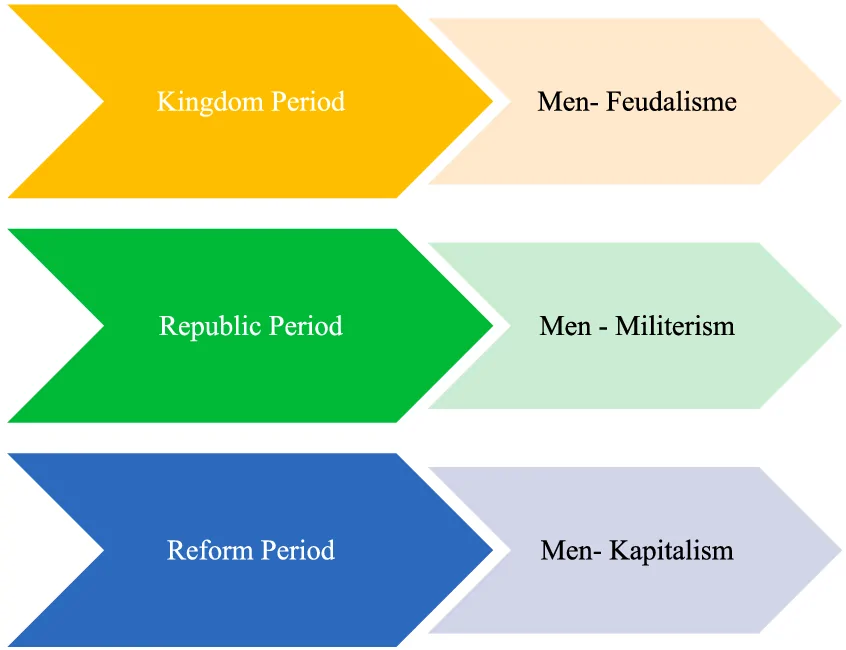
Configuration of political actors.

Moreover, this becomes even more significant when the configuration schemes appear in layers. For example, a configuration might be a man who is also a noble, a man who is also military personnel, or, finally, a man who is also a capital owner. This is even more significant if a configuration accumulates more than one form, such as a man who is a noble and simultaneously a capital owner. The more layers of capital configured, the stronger their political chances in the contestation arena. Conversely, even if someone is a man but lacks these forms of capital, they still experience disparities in political access.

The reproduction of power has perpetuated male domination across all historical periods examined. This has implications for the political feminization project designed for the future. The challenge lies on two fronts: first, deconstructing and challenging the prevailing hegemonic masculine formation; and second, actively building and supporting women from various classes, including men outside the hegemonic masculine mold, youth, and elements of civil society who share an interest in changing the unequal configuration of power (Gramsci, 1971; Carastathis, 2014).

This research has demonstrated how power transforms and adapts to changing times. The determining domains of each period (nobility, military, and capital owners) have consistently maintained their patriarchal core. This is a historical endeavor in which efforts to break this cycle of power remain unfinished, while efforts to build a foundation for new political legitimacy based on equality and social justice, free from the traps of oppressive intersecting identities, continue. Ultimately, the author realizes that the success of political feminization will depend largely on the ability to build a more solid coalition and develop a collective movement that transcends class, gender, or specific groups.

### Cultural barriers I: Siri’ and the disciplining of female bodies

Beyond structural barriers, cultural discourses function as what Foucault terms “technologies of the self,” practices through which individuals constitute themselves according to prevailing norms. The Bugis value of siri’ (honor/shame) emerged as the most powerful cultural mechanism regulating women’s political participation.

Puang Matoa (60), a cultural figure and leader of the Bissu community, explained:

“Women are the symbol of the family’s siri’; they are highly valued and guarded in such a way. Therefore, every family is obliged to protect its daughters. Protecting daughters is the same as protecting the family’s siri’ and dignity. The family’s dignity lies in the nobility (alebbirengna] of its women)”.

This testimony operationalizes Foucault’s concept of biopower: women’s bodies become the repository of collective honor. The statement is not merely descriptive but performative; it produces the very reality it describes. Women learn to experience their bodies not as their own but as symbols of family honor. This is power at its most effective: operating through self-regulation rather than external coercion (Foucault, 1977). Young cultural observer Abdi Mahesa (28) elaborated on the spatial dimensions of this discourse:

“Women are termed like ‘Lise kowari or lise jajareng’ meaning ‘contents of the room.’ This meaning is very identical to domestic work, especially the reproductive function of the husband-wife relationship. Women are also understood as ‘Awi or pakkawi’ meaning a sarong or cloth that covers the body. Symbolically, it can be understood that women are markers of honor that must be preserved and guarded, by themselves as ‘pakkawi’ or by their families for the sake of the family’s good name. Conversely, men are also largely understood as the protectors of that ‘awi’”.

This spatial lexicon is a discursive apparatus that produces gendered space. Following Massey (1994), space is not neutral but actively produced through power relations. The metaphor “contents of the room” constructs women’s legitimate space as interior and private; men as “protectors” are legitimately mobile and public. This is not merely cultural description but a performative act that disciplines female bodies by defining where they belong (Butler, 1990). He contrasted this with masculine spatial metaphors:

“In Bugis sompe (migrating) tradition, men are prioritized as path openers who face risks. Women follow only after men succeed. Men are symbolized by three associations: Ri Congari (asked for opinions), Ri Allari (a place of refuge), and Ri Sanresi (support/protector when facing problems)”.

Prof. Safira Suhra (52) offered a nuanced interpretation of how these values are internalized:

“Bugis women greatly respect their men, and as long as there is an adult male in the house, he is the one trusted, especially for political matters. I see in the experiences of several female politicians, they only decide to run for office at various levels later when their husband or the men in their family no longer run”.

This testimony reveals the operation of what Kandiyoti (1988) terms the “patriarchal bargain”: women accept domestic and political deference to men as a source of dignity within the terms their culture provides. However, from a Foucauldian perspective, this is not merely a bargain but a technology of the self: women monitor their own political ambition, deferring to husbands and male relatives, internalizing the belief that politics is masculine terrain. The result is a self-produced political subjectivity that experiences male leadership as natural and female leadership as exceptional and therefore requiring justification (Foucault, 1988).

This observation was confirmed by Andi Singkeru Rukka (47), a cultural observer:

“There is a living principle in Bugis society, where women consider that when their husband is successful, the wife’s dignity is also elevated. Conversely, when a woman or wife is successful, there is an impression that the husband is actually considered demeaned”.

This is a crucial insight from an intersectional perspective: the siri’ discourse operates differently on men and women. For men, success elevates both self and family; for women, success threatens to demean husbands. This asymmetry is not accidental but structural; it produces a gendered double standard in political ambition. Women’s success is not celebrated but perceived as emasculating, which, in turn, becomes a reason for women to self-limit their aspirations. This is how power operates through subjectivity: women do not need to be barred from politics by external rules; they bar themselves because they have internalized the belief that their success would dishonor their men (Foucault, 1988).

These testimonies reveal how siri’ operates as a disciplinary mechanism (Foucault) that produces docile female bodies and subjects. The value is not merely an external constraint but an internalized self-regulation, a “technology of the self” through which women monitor their own behavior, aspirations, and spatial mobility. The metaphors of “contents of the room” and “covering cloth” are not innocent descriptions but discursive practices that produce the very reality they describe: women as domestic, private, and in need of protection, men as public, mobile, and protective.

### Cultural barriers II: gendered spatial disciplines and the production of political subjectivity

The spatial dimensions of cultural discourse emerged as particularly significant. A key finding from our analysis of the La Galigo epic and contemporary proverbs is the systematic mapping of gendered space:

“Akkatuongenna urane ri cappana langie, na iya allaringenna makkunraie ri olona sumpangnge”.

(The life space of men is at the edge of the sky, while the shelter of women is at the front door)

This proverb is not merely descriptive but performative (Butler, 1990). It produces the spatial reality it names: men’s legitimate space is unbounded (“edge of the sky”), while women’s legitimate space is bounded (“front door”). From a Foucauldian perspective, this operates as a spatial technology of power that disciplines bodies by defining where they belong and where they do not (Foucault, 1977).

Abdi Mahesa (28) explained the implications:

“Akkatuongenna urane ri cappana langie can be interpreted as the life and livelihood space of men being unlimited, analogized as ‘cappa’na langie’ or the edge of the sky. This signifies that men can explore a very broad, unlimited journey or space. Conversely, women are actually limited by the meaning of ‘allaringenna makkunraie ri olona sumpangnge’. ‘Ri olona sumpangnge’ means at the front door. This confirms that the construction of women’s roles is not far from the home or household environment”.

This testimony operationalizes what feminist geographer Massey (1994) calls the gendered production of space. Space is not neutral; it is actively produced through power relations. In Bone, the spatial binary of unlimited space for men and bounded space for women has concrete political implications: the political arena (government offices, campaign rallies, coffee shops as political discussion centers) is symbolically coded as masculine space. Women who enter are perceived as transgressing natural boundaries. This is how power operates at the capillary level: women do not need to be explicitly barred; they learn that public space is not for them (Foucault, 1977).

He further elaborated on naming practices:

“Men have names that show or reflect their masculine identity, such as; La Mata Esso, La Pateddungi, Walinono. La Mata Esso can be understood as a figure who illuminates, from the word Matahari (sun). La Pateddungi means shading, sheltering or protecting. While Walinono means shining. Conversely, women’s names are more identical with decoration. Epithets for female figures tend to associate with women’s ‘beauty’ that can be witnessed by the five senses, such as: ati kalepping (contents of the chamber), bēlo jajareng (decoration of the bridal bed), liseq sinarangeng (contents of the stretcher), anaq dēwata (goddess), punna bolaē (the owner of the house). All these epithets show that women are objects pleasing to the eye, pleasing to men’s hearts, easily conquered, and holders of domestic affairs”.

From an intersectional perspective (Crenshaw, 1989), this naming lexicon reveals how gender is constructed through multiple intersecting discourses. Men’s names emphasize agency, illumination, and protective qualities coded as politically valuable. Women’s names emphasize passivity, decoration, and domesticity, qualities coded as politically irrelevant. This is not innocent cultural variation but a discursive apparatus that produces gendered political subjectivity. As Foucault (1972) would argue, these naming practices are statements within a discursive formation that defines what can be said, who can speak, and what counts as legitimate political identity. A woman named “contents of the chamber” does not see herself as a potential regent; a man named “the sun who illuminates” sees himself as a natural leader. Rahmatunnair (52) noted how Islamic inheritance law intersected with these cultural values:

“Urane mallempa (men carry), makkunrai ma’jujung (women bear on head) this is commonly used as the basis for inheritance division. It means men receive two portions and women receive one portion. This strengthened only after the arrival of Islam. Previously, the division was very equal, and there are still many who apply it today. For example, land division was simply divided according to the amount of land and number of children”.

This testimony is analytically crucial for two reasons. First, it demonstrates Foucault’s concept of discontinuity within continuity: the patriarchal interpretation of inheritance law (men receiving twice women’s share) is not an ancient tradition but a relatively recent accretion that came with Islam. Second, it reveals what we call discursive layering: different historical discourses (pre-Islamic Bugis, Islamic, colonial, and post-colonial) coexist and interact. Crucially, this opens space for what we term discursive re-articulation: because the more patriarchal interpretation is historically layered rather than original, it can be contested from within the tradition itself. The testimony that “previously, the division was very equal” provides a counter-narrative resource for feminist reinterpretation. From an intersectional perspective, this shows that religious and cultural discourses are not monolithic but carry within them both patriarchal accretions and egalitarian possibilities (Collins and Bilge, 2020).

This finding is crucial: it demonstrates that cultural and religious discourses are not static but historically layered. The more patriarchal interpretation of inheritance law (men receive twice women’s share) is a relatively recent accretion, not an ancient tradition. This opens space for what we term discursive re-articulation: the possibility of reclaiming more egalitarian interpretations from within the cultural tradition itself.

### Cultural barriers III: Siri’ as Technology of the Self and the production of patriarchal subjectivity

The effectiveness of siri’ as a regulatory mechanism lies in its internalization. As Foucault’s concept of technologies of the self suggests, power operates most effectively when it is not experienced as external coercion but as self-evident truth. Young activist Amar (21) articulated this during a focus group discussion:

“Historically, women haven’t gotten much space for self-development; their placement in the domestic sphere has implications for their opportunities to access and develop themselves, including building socio-political networks. I want to quote Mohammad Hatta: If you educate one man, you educate one person, but if you educate one woman, you educate a generation”.

This testimony reveals the internalized dimension of gendered exclusion. Amar’s use of Hatta’s quote is telling: it frames women’s political absence as a loss of developmental potential for the next generation, not as a matter of women’s rights or justice. From a Foucauldian perspective, this is how hegemonic discourse operates: it does not need to explicitly exclude women; it redefines the terms of inclusion so that women’s value is located in reproductive rather than political labor. Women are educated not to lead but to educate future generations. This is a technology of the self that women internalize as aspiration rather than limitation (Foucault, 1988). Political observer Supriadi (49) noted the subjective dimension:

“Besides that, most women feel it’s enough to just be at home taking care of household matters. Women who are active in public spaces also usually feel it’s enough once they have a job with a steady income and don’t need to obsess about becoming officials. This shows the low obsession of most women to enter the political world”.

This testimony could easily be read as victim-blaming women for lacking ambition. However, from a Foucauldian perspective, what Supriadi calls “low obsession” is actually the successful operation of power as self-regulation. Women do not need to be barred from politics by formal rules; they have internalized the belief that politics is not for them, that domestic work is sufficient, that a steady job is “enough.” This is power at its most insidious: it produces subjects who experience their own exclusion as contentment (Foucault, 1977). From an intersectional perspective (Crenshaw, 1989), this internalization is not uniform; noble women may feel pressure to maintain family honor through domesticity, while commoner women may feel that politics is for the elite. The “low obsession” is an intersectional product, not a universal female trait.

However, young activist Zhera Sarnia (17) offered a critical counter-perspective during an FGD:

“Women’s issues are still often considered not yet worthy, including from their closest environment and even among their own circles. But it’s unfair if we only suspect and question women. Are all men also all worthy of being leaders? Including those currently holding strategic positions in politics and government?”

The testimonies above reveal that cultural discourses are not monolithic or static; they are historically layered, carrying within them both patriarchal accretions and egalitarian possibilities, as seen in the intersection of Bugis adat and Islamic inheritance law. To fully grasp how these discourses translate into lived experience and political subjectivity, we must move beyond analyzing their content to examining their systematic structure and internalization. Table 2 provides a comprehensive inventory of the cultural lexicon, metaphors, epithets, and conceptual associations that construct and regulate gendered subjectivities in Bugis Bone society. Drawing from the La Galigo epic, traditional proverbs (pappaseng), and interviews with cultural informants, the table illuminates the discursive mechanisms through which gender difference is produced, naturalized, and hierarchically ordered. As we will demonstrate in the subsequent section, the power of these discourses lies not merely in their existence but in their operation as “technologies of the self” (Foucault, 1988), internalized norms through which women monitor their own bodies, aspirations, and spatial mobility, producing the very patriarchal subjectivity that appears to them as self-evident truth.

From a theoretical synthesis perspective, what we have documented in this section is the operation of siri’ as a triple mechanism: (1) as external discourse (proverbs, naming practices, spatial metaphors), (2) as internalized technology of the self (women deferring to men, feeling “enough” with domestic work), and (3) as intersectional filter (operating differently for noble *versus* commoner women, for women with *versus* without political families). This triple operation—discursive, subjective, and intersectional—explains why formal democratic reforms (quotas, elections) have failed to produce a single female regent candidate in Bone. Power operates not only at the level of institutions but at the level of bodies, desires, and subjectivities (Foucault, 1977; Crenshaw, 1989).

Table 2 presents a systematic inventory of cultural discourses, metaphors, epithets, and conceptual associations that construct and regulate gendered subjectivities in Bugis Bone society. Drawing from analysis of the La Galigo epic, traditional proverbs (pappaseng), and interview data with cultural informants, this table illuminates the discursive mechanisms through which gender difference is produced, naturalized, and hierarchically ordered. The table organizes cultural terms according to gender, providing translation, semantic meaning, attributed characteristics, gender preferences, practical spatial relevance, and discursive function as a power mechanism.

The cultural lexicon for women reveals a consistent pattern of spatial restriction and domestic association. The term Lise Kowari (room contents) explicitly constructs women as inhabitants of interior spaces, their identity coterminous with the domestic realm. This metaphor operates as what Foucault terms a disciplinary spatialization: the assignment of bodies to specific spaces as a mechanism of social control (Foucault, 1977). Similarly, Ri olona Sumpangnge (at the front door) literally limits women’s legitimate mobility to the threshold of the home, encoding what Young identifies as the patriarchal regulation of women’s spatial range (Young, 2020).

The value Malabbi (unpretentious, friendly, polite) presents an apparent paradox: it associates women with positive moral qualities while simultaneously linking these qualities to restricted mobility; the less they leave the house, the more they are identified with malabbi. This reveals what Kandiyoti terms the patriarchal bargain: women gain social esteem through conformity to spatial constraints that simultaneously limit their access to public power (Kandiyoti, 1988). The characteristic attributed to Malabbi (Passive–Active) and its practical relevance (Masculine–Domestic–Public) suggest this term operates at the boundary between feminine virtue and masculine-coded public action, though ultimately reinforcing domestic placement.

Malampe Nawa-nawa (has a good memory) associates women with cognitive capacity, but this capacity is directed toward organizing the contents of the house. Even when women are attributed positive qualities, these qualities are domesticated; their exercise is confined to the private sphere. This exemplifies what Bourdieu would identify as the symbolic violence of gender classification: women’s virtues are recognized but channeled into subordinate roles (Bourdieu, 1977).

The term Mancujung (uphold/carry burdens) is particularly significant. While it acknowledges women’s labor and strength, its practical relevance is categorized as Public–Marginal, suggesting that women’s work, even when publicly visible, is rendered peripheral or supplementary to men’s public roles. This reflects what Connell identifies as the gender order’s capacity to acknowledge women’s contributions while maintaining their subordination (Connell, 1995).

In striking contrast, the cultural lexicon for men constructs unbounded spatial mobility and institutional authority. Ri Cappa’na Linoe (at the end of the world) grants men unlimited geographical range, directly opposing the spatial confinement encoded in women’s terms. This metaphor produces what Massey terms masculine spatiality: the construction of public space as men’s natural domain (Massey, 1994).

The terms Ri Congari (can be asked for opinions), Ri Sanresi (responsible), La Mata Esso (the Sun), and La Pateddungi (shading) collectively construct men as sources of knowledge, protection, authority, and enlightenment. These are not merely descriptive but performative (Butler, 1990): they produce the reality they describe, shaping how men understand themselves and their social possibilities. The consistent attribution of “Active” and “Masculine” characteristics, with practical relevance spanning “Domestic–Dominant–Public,” constructs men as legitimate actors across all social spheres.

Passompe (migrant worker) is particularly significant, encoding the expectation that men will leave home to seek a livelihood, a mobility explicitly denied to women. This constructs what Connell terms hegemonic masculinity: men as breadwinners, risk-takers, and public actors whose legitimacy derives from their capacity to operate beyond domestic space (Connell, 1995).

### The “Kosong-kosong” phenomenon: informal agency amid executive exclusion

While the structural and cultural barriers in Bone produce a near-total exclusion of women from formal executive positions, our findings also reveal a parallel, informal domain of power locally known as kosong-kosong. This term describes the “behind-the-scenes” influence wielded by women, particularly those from noble or political families, over the decision-making processes of their husbands, fathers, or brothers who hold formal office. As an NGO activist (55) noted:

“Do not be mistaken; while the Regent’s chair is occupied by a man, the ‘direction of the wind’ is often determined at the dinner table. We call it ‘kosong-kosong’ it is an empty space in the formal structure, but it is filled with the whispers and strategic advice of wives and mothers. They are the unofficial kingmakers who manage patronage networks and family siri’ from the domestic sphere.”

This phenomenon represents a specific form of feminine agency within the constraints of intersectional masculinism. From a Foucauldian perspective, kosong-kosong functions as a counter-conduct, a way for women to exercise power without transgressing the spatial boundaries of the “front door” (ri olona sumpangnge). However, as our intersectional matrix suggests, this agency is highly classed; it is a privilege primarily available to noble women (Anak Arung) who possess the social capital to influence elite networks. Although kosong-kosong allows for an “informal feminization of influence,” it simultaneously reinforces the formal exclusion of women by normalizing their presence only as “hidden advisors” rather than legitimate public leaders. This confirms that even when women exercise power, they do so in ways that do not challenge the patriarchal core of the executive monopoly.

## Discussion

### Theorizing intersectional masculinism: power, discourse, and the durability of male political dominance

This section synthesizes Foucault’s analytics of power with Crenshaw’s intersectionality to advance our core theoretical contribution: the concept of intersectional masculinism. We organize the discussion around four interconnected themes: discontinuity without transformation, siri’ as an intersectional technology of the self, the spatial production of masculine political subjectivity, and intersectional masculinism as an adaptive configuration of power.

### Theorizing intersectional masculinism: definition, distinction, and adaptive mechanisms

This study advances the concept of intersectional masculinism as its primary theoretical contribution. Defined formally, intersectional masculinism refers to the adaptive configuration of privilege-conferring intersections of lineage, military affiliation, and economic capital that preserves male executive monopoly across regime changes by reconfiguring rather than dismantling patriarchal power. This concept extends beyond static patriarchy frameworks by capturing how masculine dominance persists through transformation rather than stasis.

### To distinguish intersectional masculinism from established concepts in the literature, three key differences must be articulated

First, unlike hegemonic masculinity (Connell, 1995, 2005), which emphasizes a culturally idealized form of male dominance that undergoes slow, incremental transformation, intersectional masculinism captures the adaptive and predatory nature of male dominance in transitional societies. Hegemonic masculinity presupposes a relatively stable cultural ideal; intersectional masculinism posits that masculine hegemony is not a fixed archetype but a fluid configuration that maintains its patriarchal core by strategically shifting between different privilege-conferring intersections. As our genealogical analysis shows, the “ideal” masculine leader in Bone shifted from the noble-aristocrat (kingdom era) to the military-bureaucrat (New Order) to the capitalist-political entrepreneur (Reform era), yet the gendered outcome of absolute male executive monopoly remained constant.

Second, unlike hybrid masculinities (Bridges and Pascoe, 2014), which focus on men’s discursive “softening” of dominance through selective incorporation of marginalized masculinities, intersectional masculinism attends to the cumulative layering of material and symbolic capitals such as noble lineage, military affiliation, and economic capital that operate as entry barriers. Hybrid masculinities focus on cultural performance and the ways men appropriate marginalized identities to appear less dominant; intersectional masculinism focuses on structural access and the ways privilege-conferring intersections accumulate to create impenetrable barriers. In Bone, a man cannot access executive power without these material capitals, and a woman with all of them still cannot.

Third, while both hegemonic masculinity and hybrid masculinities treat change as gradual or elastic accommodation, intersectional masculinism introduces the concept of discontinuity without transformation: the pattern wherein political form changes radically (kingdom → republic → democracy) while masculine outcomes remain constant (absolute male executive monopoly). This captures a phenomenon that neither Connell’s nor Bridges and Pascoe’s frameworks can explain: regime changes that promise transformation yet reproduce the same gendered outcome through different configurations of power.

### The adaptive mechanisms of intersectional masculinism operate through three distinct configurations across bone’s history

1. Feudal-Aristocratic Masculinity (Kingdom Era, 14th–20th Century): Power required noble lineage (anak arung) and spiritual charisma. Women could access leadership only at the intersection of gender and aristocratic class, an exception that reinforced rather than dismantled feudal hierarchy. As cultural figure Andi Singkeru Rukka explained: “In Bone’s tradition, leadership attaches only to blue blood. When a woman becomes Arung, it is first because she is ‘Anak Arung’ [child of a ruler], and only second because of personal capacity.” This is not “gender equality” but a class-privileged exception that proves the rule. The presence of six female rulers in the kingdom era does not indicate openness to women’s leadership; it indicates that class could temporarily override gender for a tiny elite, while the vast majority of women commoners remained excluded. From an intersectional perspective, this reveals how gender and class intersected to produce “discrimination within discrimination” (Crenshaw, 1989): noble women accessed power, but commoner women were excluded entirely.
2. Military-Bureaucratic Masculinity (New Order, 1966–1998): Power required male gender, military affiliation (ABRI/TNI), and state loyalty. This configuration systematically excluded all women, since the military was an almost exclusively male institution. As political observer Supriadi noted: “Historically, in the New Order era, the rulers prioritized figures from the military to occupy regional head positions. And we know they were, by definition, men.” Critically, this did not replace feudal-aristocratic masculinity but merged with it: military officers sent to Bone often had or strategically acquired genealogical ties to the aristocracy. This created a hybrid elite in which legitimate power required the intersection of male gender, military affiliation, and noble lineage. This intersection effectively excluded all women, since the military was a male-only institution, and even noble women could not compensate for their gender with lineage alone.
3. Capitalist-Electoral Masculinity (Reform Era, 1999–Present): Power requires either male gender or female gender with family succession, plus economic capital (≥Rp15 billion) and patronage networks. Journalist Abd. Muhaimin explained: “Just winning a district legislative candidacy requires funds up to billions [of rupiah], let alone running for regent. Current contests are very thick with money politics.” From an intersectional perspective, this creates a systematic barrier for women, who historically have less access to economic capital than men. The 15 billion rupiah threshold operates as an intersectional filter: only those at the intersection of male gender (or female gender with family wealth) and economic capital can enter the contest. This creates a sharp boundary: a man without noble lineage or capital cannot access power, but a woman with both still cannot. Gender trumps other privileges for executive exclusion, even when those privileges enable legislative inclusion.

Crucially, these configurations are not sequential replacements but layered and cumulative. Contemporary candidates must simultaneously possess noble lineage (feudal capital), military or bureaucratic connections (institutional capital), and massive financial resources (economic capital). This layering creates an intersectional barrier that few women can penetrate. This is the signature pattern of intersectional masculinism: form changes, monopoly persists; discontinuity without transformation.

### Siri’ as an intersectional technology of the self: operationalizing Foucault–Crenshaw integration

This section demonstrates how the integration of Foucault and Crenshaw introduced in the theoretical framework is operationalized to analyze empirical findings. The integration is not merely a theoretical assertion but an analytical practice: every finding is examined through (1) Foucauldian attention to discourse, self-regulation, and spatial discipline, and (2) Crenshawian attention to differential operation across social positions.

The Bugis value of siri’ (honor/shame) functions as what Foucault terms a technology of the self (Foucault, 1988), a practice through which individuals actively constitute themselves according to prevailing norms, experiencing power not as external coercion but as self-evident truth. Our analysis demonstrates that siri’ operates through a triple mechanism:

First, siri’ operates as external discourse (Foucault’s discursive formation). Siri’ circulates through proverbs, naming practices, and spatial metaphors that systematically associate women with domesticity and men with unbounded mobility. The proverb “Akkatuongenna urane ri cappana langie, na iya allaringenna makkunraie ri olona sumpangnge” (“men’s life space is at the edge of the sky, women’s shelter is at the front door”) is not merely descriptive but performative (Butler, 1990): it produces the spatial reality it names. Men’s names emphasize agency, illumination, and protective qualities coded as politically valuable; women’s names emphasize passivity, decoration, and domesticity qualities coded as politically irrelevant. Cultural observer Abdi Mahesa explained: “Men have names that show their masculine identity, such as La Mata Esso (the sun who illuminates), La Pateddungi (shading/protecting). Women’s names are more identical with decoration: ati kalepping (contents of the chamber), liseq sinarangeng (contents of the stretcher).” From a Foucauldian perspective, these naming practices are statements within a discursive formation that defines what can be said, who can speak, and what counts as legitimate political identity.

Second, siri’ operates as internalized self-regulation (Foucault’s technology of the self). Women do not need to be barred from politics by formal rules; they have internalized the belief that politics is not for them, that domestic work is sufficient, that a steady job is “enough.” As political observer Supriadi noted: “Most women feel it’s enough to just be at home taking care of household matters. This shows the low obsession of most women to enter the political world.” From a Foucauldian perspective, what Supriadi calls “low obsession” is actually the successful operation of power as self-regulation, power at its most insidious, producing subjects who experience their own exclusion as contentment. This was confirmed by Andi Singkeru Rukka: “There is a living principle in Bugis society, where women consider that when their husband is successful, the wife’s dignity is also elevated. Conversely, when a woman or wife is successful, there is an impression that the husband is actually considered demeaned.” This is a crucial insight: for men, success elevates both self and family; for women, success threatens to demean husbands. Women bar themselves from politics because they have internalized the belief that their success would dishonor their men.

Third, siri’ operates as an intersectional filter (Crenshaw’s structural intersectionality). The same siri’ discourse does not operate identically for all women. Noble women experience siri’ as a duty to maintain family honor through domesticity and political deference; commoner women experience it as a barrier to economic mobility and public presence. Women from political families (to mapparenta) experience siri’ as an obligation to continue the family legacy through managed inclusion; women without such families experience it as complete exclusion. This differential operation is precisely what Crenshaw (1989) terms “discrimination within discrimination”: the intersectional invisibility of multiply marginalized women. As our intersectional matrix demonstrated, women at the intersection of non-noble class, non-political lineage, and female gender face compounded barriers that cannot be explained by gender alone. The experiences of female politicians like Andi Tenri Walinonong, who succeeded because she sat at the intersection of gender, lineage (political family with multiple village heads), and social capital, confirm that women can access legislative power when, and only when, they possess other privilege-conferring attributes that compensate for their gender.

The integration of Foucault and Crenshaw thus enables us to see siri’ not merely as a cultural value but as an intersectional technology of power: a mechanism that simultaneously produces gendered subjects through discourse (Foucault), differentiates those subjects across social positions (Crenshaw), and adapts across historical discontinuities while maintaining patriarchal outcomes (discontinuity without transformation).

This triple mechanism—discursive, subjective, and intersectional—explains why formal democratic reforms (quotas, elections) have failed to produce a single female regent candidate in Bone. Power operates not only at the level of institutions but at the level of bodies, desires, and subjectivities (Foucault, 1977; Crenshaw, 1989). The most effective barrier is the one women carry inside themselves. Siri’ works not because men enforce it, but because women believe it.

### Discontinuity without transformation: bridging Foucault and historical analysis

Foucault’s concept of discontinuity (1972) provides the analytical tool to trace how power reconfigures across regime changes. Our findings reveal a striking pattern: despite radical discontinuities in political form from kingdom to republic, from authoritarianism to democracy, male monopoly over executive power in Bone has persisted unbroken for over seven decades. We term this discontinuity without transformation.

This finding complicates Foucault’s own framework. In Foucault, discontinuities typically mark epistemic ruptures where regimes of truth fundamentally shift. In Bone, however, we find discontinuities in legitimating principles (noble lineage → military loyalty → electoral victory) but continuity in gendered outcomes (absolute male executive monopoly). This suggests that Foucault’s analytics, while essential for tracing discursive shifts, require intersectional specification (Crenshaw) to explain why some hierarchies persist across epistemic ruptures.

The integration works as follows: Foucault’s discontinuity identifies how power reconfigures; Crenshaw’s intersectionality explains which intersections matter in each configuration; and intersectional masculinism specifies how these intersections combine to maintain male dominance. This layered analytical approach—discontinuity (Foucault) + intersectional matrix (Crenshaw) + adaptive configurations (intersectional masculinism)—enables us to explain the “Bone Paradox”: how the same structural outcome (absolute executive exclusion) can be reproduced through entirely different legitimating principles across radical political ruptures.

The discontinuities we identified are not merely descriptive periodization but analytical evidence of intersectional masculinism in operation.

### The spatial production of masculine political subjectivity

Foucault’s concept of disciplinary spatialization (1977) and Crenshaw’s intersectional sensitivity together explain the spatial dimensions of exclusion. The systematic mapping of gendered space—men at the edge of the sky, women at the front door—has concrete political implications. Following feminist geographer Massey (1994), space is not neutral but actively produced through power relations. In Bone, the political arena (government offices, parliamentary buildings, coffee shops as political discussion centers) is symbolically coded as masculine space. Women who enter are perceived as transgressing natural boundaries, not because of formal rules but because of deeply embedded spatial norms.

This operates through what Fincher and Iveson (2017) term invisible spatial boundaries: norms and practices that systematically limit women’s agency in the public realm. Cultural observer Abdi Mahesa explained: “Women are termed like ‘Lise kowari or lise jaijareng’ meaning ‘contents of the room.’ This meaning is very identical to domestic work. Conversely, men are prioritized as path openers who face risks.” The spatial binary—unlimited for men, bounded for women—has concrete political implications: women learn that public space is not for them; men learn that public space is their natural domain.

The concept of kosong-kosong offers a counterpoint: women’s influence operates through behind-the-scenes categories. NGO activist (55) explained: “Do not be mistaken; while the Regent’s chair is occupied by a man, the ‘direction of the wind’ is often determined at the dinner table. We call it ‘kosong-kosong’ it is an empty space in the formal structure, but it is filled with the whispers and strategic advice of wives and mothers.” From a Foucauldian perspective, kosong-kosong functions as a counter-conduct, a way for women to exercise power without transgressing spatial boundaries. From an intersectional perspective, this agency is highly classed and primarily available to noble women (anak arung) who possess the social capital to influence elite networks. Intersectional masculinism thus explains not only exclusion but also the specific forms of “inclusion” available to privileged women: informal influence without formal power.

This reveals a crucial gendered double standard: men’s political ambition is normalized as natural; women’s political ambition is exceptionalized as requiring justification. This double standard is not accidental but structural; it is built into the siri’ discourse and its spatial logic. Politics in Bone is not just dominated by men; it is spatially coded as masculine. Women do not need to be barred; they learn that the public square is not for them.

### Implications for the global south: toward a transferable framework

The intersectional masculinism framework offers analytical leverage beyond Bone for understanding women’s executive exclusion in other Global South contexts where democratic institutions coexist with patriarchal cultural codes. Our finding of “discontinuity without transformation”—political regimes shift, but male monopoly persists—finds resonance in honor-shame codes across diverse contexts: izzat in Pakistan, maratabat in the Philippines, and namus in Turkey.

In contexts governed by izzat or namus, women’s bodies are constructed as repositories of collective honor, mirroring siri’s function in Bone. In Pakistan, while women have achieved national leadership (Benazir Bhutto as Prime Minister), sub-national executive exclusion remains high because izzat defines the public sphere as a risk to female honor, the same logic that confines Bugis women to “the front door.” Similarly, maratabat among the Moro people in the Southern Philippines illustrates how clan honor is intricately tied to male dominance within a political economy of patronage and capital, mirroring the feudal-aristocratic configuration of Bone’s kingdom era.

What intersectional masculinism contributes to comparative analysis is the specification of mechanisms: (1) honor-shame codes operate as technologies of the self, internalized rather than externally enforced; (2) they are intersectionally differentiated, operating differently for elite *versus* non-elite women; and (3) they adapt across regime changes, shifting which intersections matter (lineage → military → capital). This suggests that democratic transitions alone cannot dismantle gendered monopolies if the underlying intersectional barriers remain unchallenged.

By identifying the pattern of “discontinuity without transformation,” this study offers a transferable framework for understanding durable political exclusion across the Global South from Pakistan’s izzat to the Philippines’ maratabat and Turkey’s namus. In each context, the question is not whether democratic transitions have occurred but whether the underlying intersectional configurations (feudal, military, capitalist) have been reconfigured in ways that actually enable women’s access to executive power or merely reproduce male dominance in new forms.

### Practical implications

Based on the findings and analysis, four targeted strategies are required. First, policymakers should deconstruct siri’ as a technology of the self by mobilizing counter-narratives, the historical memory of six female rulers, and pre-Islamic egalitarian inheritance practices to show that women’s political leadership is neither alien nor dishonoring. Second, policymakers should support women’s organizing across class and lineage divisions, not only within elite circles, because barriers operate at the intersection of gender with class and family background. Third, policymakers should engage men as feminist allies by reframing masculinity away from honor-based protection toward shared political responsibility. Fourth, policymakers should reduce the cost of politics through public financing mechanisms that specifically support women candidates from non-elite backgrounds, as the current 15 billion rupiah threshold for regent campaigns systematically excludes women without dynastic wealth. Gender quotas alone are insufficient. Effective intervention must dismantle intersectional barriers where gender meets class, lineage, and capital.

## Conclusion

This study demonstrates that women’s political marginalization in Bone Regency is not a product of static patriarchy but of intersectional masculinism, an adaptive configuration of privilege-conferring intersections (noble lineage, military affiliation, and economic capital) that preserves male executive monopoly across regime changes. By synthesizing Foucault’s discontinuity with Crenshaw’s intersectionality, we introduce “discontinuity without transformation” as a conceptual tool explaining how masculine power reconfigures rather than collapses under democratic transitions, extending hegemonic masculinity theory beyond static cultural ideals. This study directly addresses the three research gaps identified in the introduction. First, it fills the executive gap by shifting analytical focus from legislative quotas to the executive sphere, demonstrating how an absolute male executive monopoly persists despite women’s legislative representation. Second, it bridges the historical gap by tracing the reconfiguration of masculine dominance across three distinct political eras, revealing the pattern of “discontinuity without transformation.” Third, it closes the discursive-intersectional gap by analyzing siri’ as an intersectional technology of the self that operates differentially across class and lineage, thereby connecting cultural discourse to political exclusion. By doing so, the study not only answers the research questions but also provides a transferable framework for understanding durable gendered political exclusion in the Global South. Three findings substantiate this argument. First, social barriers operate through historical discontinuity without transformation: each regime shift from kingdom to New Order to Reform produced new legitimating principles (lineage → military → capital) while maintaining absolute male executive monopoly. Second, cultural barriers operate through siri’ as an intersectional technology of the self, a triple mechanism (discursive, subjective, intersectional) that disciplines female bodies through spatial confinement, internalized self-regulation, and differential operation across class and lineage. Third, these mechanisms are intersectional: exclusion operates at the intersection of gender with class, lineage, and economic capital, meaning gender alone cannot explain why women from noble or political families may access legislative power but remain excluded from the executive. In policy terms, gender quotas alone are insufficient. Effective intervention requires dismantling intersectional barriers through four strategies: (1) counter-narrative mobilization reclaiming historical female leadership to deconstruct siri’ as internalized constraint; (2) cross-class women’s organizing beyond elite circles; (3) engaging men as feminist allies by reframing masculinity away from honor-based protection; and (4) public financing mechanisms to reduce the prohibitive cost of politics (currently Rp15 billion for regent campaigns). Future research should apply the intersectional masculinism framework comparatively to other Global South contexts: Pakistan’s izzat, the Philippines’ maratabat, and Turkey’s namus, to test whether honor-shame codes universally operate as technologies of the self in producing durable women’s executive exclusion. Such comparative work would further specify how intersectional masculinism adapts across different cultural and political configurations, contributing to a global theory of gendered political exclusion.

## Data Availability

The raw data supporting the conclusions of this article will be made available by the authors, without undue reservation.
